# Metabolomic and lipidomic profiling reveals distinct subtypes of glioblastoma IDH-wildtype and shared metabolic features with astrocytoma IDH-mutant grade 4

**DOI:** 10.1093/noajnl/vdag199

**Published:** 2026-09-02

**Authors:** John Paul Aboubechara, Yin Liu, Oliver Fiehn, Muhammad Sulman, Alexander Del Bosque, Diego Cantillo, Lina A Dahabiyeh, Ruben Fragoso, Jonathan W Riess, Rawad Hodeify, Orin Bloch, Vihar Patel, Orwa Aboud

**Affiliations:** Department of Neurology, University of California, Davis, Sacramento; Comprehensive Cancer Center, University of California, Davis, Sacramento; Department of Neurology, University of California, Davis, Sacramento; Department of Ophthalmology, University of California, Davis, Sacramento; West Coast Metabolomics Center, University of California, Davis, Davis; Department of Neurology, University of California, Davis, Sacramento; Department of Neurology, University of California, Davis, Sacramento; Department of Neurology, University of California, Davis, Sacramento; West Coast Metabolomics Center, University of California, Davis, Davis; Department of Pharmaceutical Sciences, School of Pharmacy, The University of Jordan, Amman, Amman Governorate, Jordan; Department of Radiation Oncology, University of California, Davis, Sacramento; Department of Internal Medicine, Division of Hematology and Oncology, University of California, Davis, Sacramento; Department of Biotechnology, School of Arts and Sciences, American University of Ras AlKhaimah, Ras AlKhaimah, United Arab Emirates; Department of Neurological Surgery, University of California, Davis, Sacramento; Department of Pathology, University of California, Davis, Sacramento; Department of Neurology, University of California, Davis, Sacramento; Comprehensive Cancer Center, University of California, Davis, Sacramento; Department of Neurological Surgery, University of California, Davis, Sacramento

**Keywords:** astrocytoma, glioblastoma, isocitrate dehydrogenase (IDH), lipidomics, machine learning, metabolomics

## Abstract

**Background:**

Glioblastoma IDH-wildtype (GBM) is a metabolically diverse and aggressive brain tumor. While transcriptomic subtypes are well-defined, they lack a direct understanding of the tumor’s metabolism. We used integrated, untargeted metabolomics and lipidomics to define the functional metabolic landscape of GBM and astrocytoma IDH-mutant, grade 4, and to identify intrinsic metabolic subtypes with specific vulnerabilities.

**Methods:**

Brain tissue from GBM patients (*n* = 38), astrocytoma IDH-mutant, grade 4 (*n* = 5), and non-neoplastic controls (*n* = 20) underwent untargeted metabolomic and lipidomic profiling. Data were analyzed using Partial Least Squares Discriminant Analysis (PLS-DA), Principal Component Analysis (PCA), and K-means clustering. Quantitative pathway enrichment analyses were performed based on established databases, and Z-scores are reported for effect sizes. A multinomial logistic regression classifier was trained to assess diagnostic utility.

**Results:**

GBM and IDH-mutant astrocytoma, grade 4, exhibited distinct metabolomic profiles, driven by 2-hydroxyglutarate accumulation in IDH-mutants. Machine learning classification trained on the metabolomic profiles achieved 95% diagnostic accuracy. Within GBM, K-means clustering of the metabolomics dataset revealed three functionally distinct subtypes. Cluster 1 was defined by fatty acid turnover. Cluster 2 was characterized by the robust accumulation of triglycerides, alongside a significantly reduced hexosylceramide-to-ceramide ratio. Cluster 3 was distinguished by upregulated one-carbon and folate metabolism, a significant depletion of acylcarnitines, and susceptibility to ferroptotic cell death.

**Conclusions:**

Metabolomic and lipidomic profiling holds promise as an accurate diagnostic tool, while also enabling a deeper metabolic understanding of high-grade gliomas. This approach resulted in the identification of three distinct metabolic subtypes of GBM, each possessing unique therapeutic vulnerabilities.

Key Points

- Glioblastoma has three metabolic subtypes, which are distinct from astrocytoma IDH-mutant, grade 4.
- Machine learning accurately classifies gliomas using metabolic signatures.
- Metabolic profiling identifies new therapeutic targets for high-grade glioma.

Importance of the Study

While the genomic and transcriptomic landscapes of glioblastoma are well-defined, the functional metabolic programs driving tumor survival remain incompletely mapped. This study utilizes integrated metabolomics and lipidomics to identify metabolic biomarkers of each tumor, while also revealing that IDH-wildtype glioblastoma segregates into three distinct functional subtypes. Unlike previous targeted studies, our comprehensive approach identifies convergent lipidomic remodeling across high-grade gliomas while exposing profound intra-tumoral heterogeneity in a number of functional characteristics. This study shifts the paradigm from genomic potential to functional action, identifying previously unrecognized, targetable metabolic vulnerabilities. These findings provide a vital foundation for developing precision metabolic interventions for patients with high-grade gliomas.

Glioblastoma IDH-wildtype (GBM) is the archetype of a recalcitrant malignancy. Despite aggressive multimodal therapy comprising maximal surgical resection, alkylating chemotherapy, and radiation, the median survival remains dismal at approximately 15 months.^1^ A primary driver of this therapeutic failure is believed to be due to the biological heterogeneity of the tumor.^2^ GBM is not a uniform cellular mass but a complex ecosystem of multiple types of cellular subpopulations, which contribute to the tumor microenvironment.^2^ Furthermore, while genomic and histological classifications remain the gold standard, instances of diagnostic ambiguity highlight the need for rapid, complementary diagnostic tools. Functional metabolic biomarkers hold significant potential in this area, yet candidate biomarkers remain largely undefined for high-grade gliomas.

Transcriptomic analyses performed as part of The Cancer Genome Atlas Network have identified three subtypes of GBM denoted as proneural, mesenchymal, and classical transcriptomic subtypes.^3^ Subsequent single-cell sequencing studies have identified four core cellular states (neural-progenitor-like, oligodendrocyte-progenitor-like, astrocyte-like, and mesenchymal-like) that drive GBM heterogeneity.^4^ Despite these breakthrough insights, there has been limited clinical application of this work at this time. This translational gap highlights a critical limitation of transcriptomics: gene expression represents cellular *potential*, whereas metabolism represents cellular *action*. In order to target the functional machinery of the tumor, it is critical to understand its active metabolic phenotype.

GBM cells exhibit remarkable metabolic plasticity, allowing them to thrive in a nutrient-deprived, hypoxic microenvironment.^5^ Beyond the classical Warburg effect, where glucose is fermented to lactate even in the presence of oxygen,^6^ glioma cells are capable of scavenging amino acids, oxidizing fatty acids, and upregulating antioxidant defenses.^7^ Gliomas have also been shown to exhibit accelerated anabolic metabolism^8^ and undergo deep systemic metabolic remodeling to support proliferation.^9^

Compounding this complexity is the role of the lipidome. Lipids are frequently dismissed as passive energy stores, yet they define the structural logic of the cancer cell.^10^ Specifically, the lipid bilayer—dictated by lipid chain length and the ratio of saturated to unsaturated fatty acids—regulates membrane fluidity, drug permeability, and receptor signaling dynamics.^10^ Furthermore, the balance of specific lipid classes, such as the ratio of monounsaturated to polyunsaturated fatty acids (MUFA/PUFA), dictates cellular susceptibility to ferroptosis, an iron-dependent form of cell death driven by lipid peroxidation.^11^ Additionally, alterations in sphingolipid metabolism, particularly the balance between ceramides and hexosylceramides, play a canonical role in mediating resistance to traditional chemotherapies.^12^ While genomic differences between GBM and IDH-mutant astrocytoma are well-characterized, the functional structural lipid landscape of these entities has rarely been compared.

In this study, we employed high-resolution untargeted metabolomics and lipidomics to dissect the functional landscape of high-grade glioma in order to identify diagnostic biomarkers while gleaning deeper insights into tumor metabolism. We identified numerous metabolites that are altered in tumors compared to controls, and we augmented the diagnostic yield of these metabolites by utilizing machine learning. We further report the identification of three distinct GBM metabolic clusters, each with unique metabolic vulnerabilities, that offer a new framework for precision metabolic oncology.

## Methods

### Patient Cohort and Sample Acquisition

Fresh-frozen brain tissue samples were obtained from patients undergoing surgical resection at the University of California, Davis. The study cohort consisted of three distinct groups: (1) GBM IDH-wildtype (*n* = 38), (2) Astrocytoma, IDH-mutant, grade 4 (*n* = 5), and (3) Non-neoplastic controls (*n* = 20). Control tissues were derived from biochemically normal brain tissue resected from patients undergoing surgery for alternative neurosurgical indications, including traumatic brain injury and epilepsy. The mean ages were 68 years for GBM IDH-wildtype, 38 years for astrocytoma IDH-mutant grade 4, and 45 years for controls. Sex distribution was 12 female and 26 male for GBM IDH-wildtype, zero females and 5 males for astrocytoma IDH-mutant grade 4, and 8 females and 12 males for controls. Ethnic distributions were 3 Hispanic, 31 White, and 4 not reported for GBM IDH-wildtype, 5 White for astrocytoma IDH-mutant grade 4, and 2 Hispanic and 18 White for controls. No survival outcomes or other tumor molecular features data were available for these patients. All diagnoses were rigorously confirmed by board-certified neuropathologists according to the *2021 WHO Classification of Tumors of the Central Nervous System.*^13^ Tissue samples were flash-frozen immediately upon resection and stored at −80°C until processing. The study was conducted in accordance with the Declaration of Helsinki and approved by the Institutional Review Board (IRB) of the University of California, Davis. Informed consent was obtained from all participants.

### Sample Preparation and Extraction

Metabolomic and lipidomic profiling was performed at the West Coast Metabolomics Center (WCMC) (Davis, CA). Samples underwent a biphasic extraction protocol to separate hydrophilic (polar metabolites) and hydrophobic (complex lipids) fractions. Briefly, frozen tissue was homogenized and extracted using a standardized solvent mixture of degassed acetonitrile, isopropanol, and water (3:3:2 v/v/v). The mixture was centrifuged to separate the supernatant, which was then divided for dual-platform analysis. The polar fraction was dried down for derivatization, while the lipid fraction was resuspended in a methanol/toluene mixture for LC-MS analysis. Prior to extraction, samples were spiked with a standardized internal standard mixture, including stable isotope-labeled primary metabolites and the SPLASH Lipidomix internal standard (Avanti Polar Lipids), to correct for extraction efficiency and instrument variance.

### Untargeted Primary Metabolomics (GC-MS)

Primary metabolite profiling was performed using Gas Chromatography-Time of Flight Mass Spectrometry (GC-TOF MS). Dried polar extracts were derivatized via methoximation (using methoxyamine hydrochloride in pyridine) followed by silylation (using N-methyl-N-trimethylsilyltrifluoroacetamide^11^). Analysis was conducted on an Agilent 5977A gas chromatograph coupled to a mass spectrometer. A generic 30 m × 0.25 mm Rtx-5Sil MS column was used with helium as the carrier gas. This platform provided broad coverage of central carbon metabolism, including glycolysis intermediates, TCA cycle acids, amino acids, sugars, and nucleosides. Metabolite identification was performed by matching mass spectra and retention indices against the in-house BinBase library and the FiehnLab database.

### Untargeted Complex Lipidomics (LC-MS)

Complex lipids were profiled using Charged Surface Hybrid (CSH) Liquid Chromatography-Quadrupole Time-of-Flight Mass Spectrometry (LC-QTOF MS). Extracts were separated on a Waters Acquity UPLC CSH C18 column (100 mm × 2.1 mm, 1.7 µm particle size) coupled to an Agilent 6530 Q-TOF mass spectrometer. Data were acquired in both positive and negative electrospray ionization (ESI) modes to maximize coverage. This workflow allowed for the speciation of major lipid classes, including phospholipids (PC, PE, PS, PI), sphingolipids (SM, Cer, HexCer), glycerolipids (TG, DG), and acylcarnitines. Lipid species were identified using the LipidBlast in silico MS/MS library, matching precursor ion mass and fragmentation patterns.

### Data Processing and Normalization

Raw data were processed to align retention times and deconvolute mass spectral peaks. To correct for systematic variation and heteroscedasticity, data were normalized to the sum of total peak intensity (mTIC) and Pareto-scaled (mean-centered and divided by the square root of the standard deviation) prior to statistical analysis. Data manipulation and pre-processing were conducted using MetaboAnalyst 6.0.

### Statistical Analysis and Clustering

All statistical analyses were performed using Python (v3.13) and MetaboAnalyst 6.0. To identify significantly altered features between groups, we performed an Analysis of Variance (ANOVA) adjusted for multiple comparisons using the Benjamini-Hochberg False Discovery Rate (FDR) method, with significance defined as an adjusted *P*-value (FDR) <.05 and a Fold Change (FC) > 2.0. Unsupervised Principal Component Analysis (PCA) was used to visualize intrinsic variance and detect outliers, while supervised Partial Least Squares Discriminant Analysis (PLS-DA) was employed to maximize separation between defined groups. Model robustness was validated using permutation testing (*n* = 1000 permutations, *P* < .001) and 5-fold cross-validation. To define metabolic subtypes, K-means clustering was applied specifically to the normalized metabolomics dataset of the IDH-wildtype GBM cohort (*k* = 3 clusters). Cluster stability was verified via PCA visualization and pairwise PERMANOVA. For quantitative lipid indices, statistical significance was assessed using the non-parametric Kruskal-Wallis test followed by Dunn’s post hoc test to account for non-normal data distributions.

### Pathway and Enrichment Analysis

Functional interpretation of the data was performed using MetaboAnalyst 6.0. Significantly altered metabolites were mapped to the Kyoto Encyclopedia of Genes and Genomes (KEGG) reference database. Relative pathway scores (Z-scores) were then calculated based on the aggregate regulation of all detected metabolites within a specific KEGG pathway relative to controls. Additionally, lipid species were grouped into classes according to the LIPID MAPS ontology, and a Lipid Class Enrichment Analysis (LCEA) was performed to calculate abundance Z-scores for each lipid class across the tumor subtypes.

### Machine Learning Classification

To evaluate the diagnostic utility of the metabolic profiles, a Multinomial Logistic Regression (MLR) classifier was developed using the Scikit-learn library (v1.6). The model utilized an L2 penalty, a regularization strength (C) of 1.0, and the L-BFGS solver to handle multiclass optimization. The model was trained on the top 20 metabolic features identified via PLS-DA variable importance in projection (VIP) scores. The dataset was stratified into a training set composed of 70% of the samples (*n* = 44; 27 GBM IDH-wildtype, 3 IDH-mutant astrocytoma grade 4, and 14 non-neoplastic controls) and an independent test set composed of the remaining 30% (*n* = 19; 11 GBM IDH-wildtype, 2 IDH-mutant astrocytoma grade 4, and 6 non-neoplastic controls). Model performance was then evaluated using a Confusion Matrix, Multi-class Receiver Operating Characteristic (ROC) analysis utilizing a One-vs-Rest strategy, and the calculation of Accuracy, Precision, Recall, and F1-Scores.

### Quantitative Metabolic and Lipidomic Indices

Quantitative indices were calculated using the normalized relative peak area abundances of the specified metabolites and lipid species to assess functional tumor biology. The glutaminolysis proxy was calculated as the ratio of glutamic acid to glutamine. An energy storage index was determined by taking the ratio of triglycerides to the sum of the structural membrane phospholipids, phosphatidylcholine and phosphatidylethanolamine. Membrane saturation was calculated as the ratio of total saturated fatty acids to total unsaturated fatty acids. The ferroptosis resistance index was defined as the ratio of monounsaturated fatty acids to polyunsaturated fatty acids. To evaluate potential drug resistance mechanisms, a marker was calculated as the ratio of hexosylceramides to ceramides. Mitochondrial stress was approximated by the total abundance of acylcarnitines, while membrane biomass was represented by the sum of the abundances of phosphatidylcholines, phosphatidylethanolamines, cholesterol, and sphingomyelins. Finally, lipid chain length was calculated as the weighted average carbon chain length of all identified fatty acyl tails.

## Results

### The Global Metabolomic and Lipidomic Landscape of High-Grade Glioma

**Figure 1.**
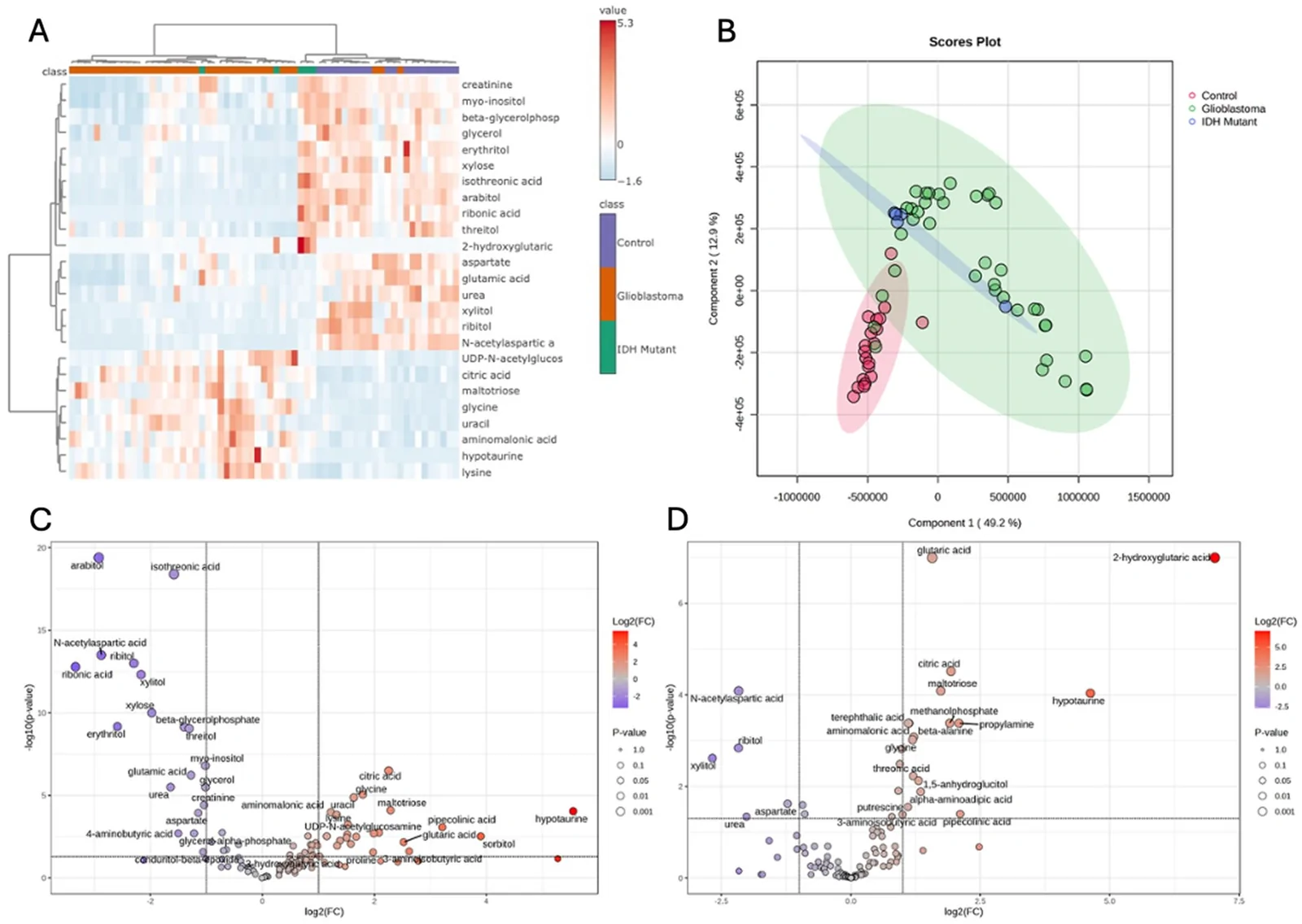
Global metabolomic landscape of high-grade gliomas. (A) Hierarchical clustering heatmap of the top 25 metabolites representing the most variance across control, GBM, and IDH mutant samples. (B) Partial Least Squares Discriminant Analysis (PLS-DA) scores plot showing distinct clustering. (C, D). Volcano plots illustrating significantly altered metabolites in GBM (C) and IDH mutant (D) tumors compared to non-neoplastic controls. Significant features (FDR < 0.05, FC > 2) include massive 2-hydroxyglutarate (2-HG) accumulation in IDH mutants and elevated hypotaurine in both GBM and IDH mutants.

Global profiling of the metabolomic and lipidomic signatures of control tissue, GBM, and IDH mutant tumors was performed. Hierarchical clustering was performed to evaluate the top 25 metabolites that best represented the variance of the broader dataset. This demonstrated distinct clustering of GBM from control tissue, while IDH mutant tumors did not cluster separately (Figure 1A). Partial least squares discriminant analysis was performed to examine the variance of the metabolites across the entire dataset from all three groups. The resulting score plot shows distinct clustering of GBM from control tissue, but IDH mutant tumors did not cluster independently (Figure 1B). This separation was found to be statistically significant, as confirmed by permutation testing (*P* < .001) and five-fold cross-validation (Figure S1). The significantly altered metabolites between GBM and control tissue are depicted in a volcano plot, with hypotaurine being amongst the metabolites increased in GBM and arabitol being decreased (Figure 1C). The significantly altered metabolites between IDH mutant tumors and control tissue are also depicted in a volcano plot, with 2-hydroxyglutaric acid demonstrating a dramatic increase in IDH and N-acetylaspartic acid being decreased (Figure 1D). The full list of significantly altered metabolites for each comparison is included in Table S1 for GBM and Table S2 for IDH-mutant.

**Figure 2.**
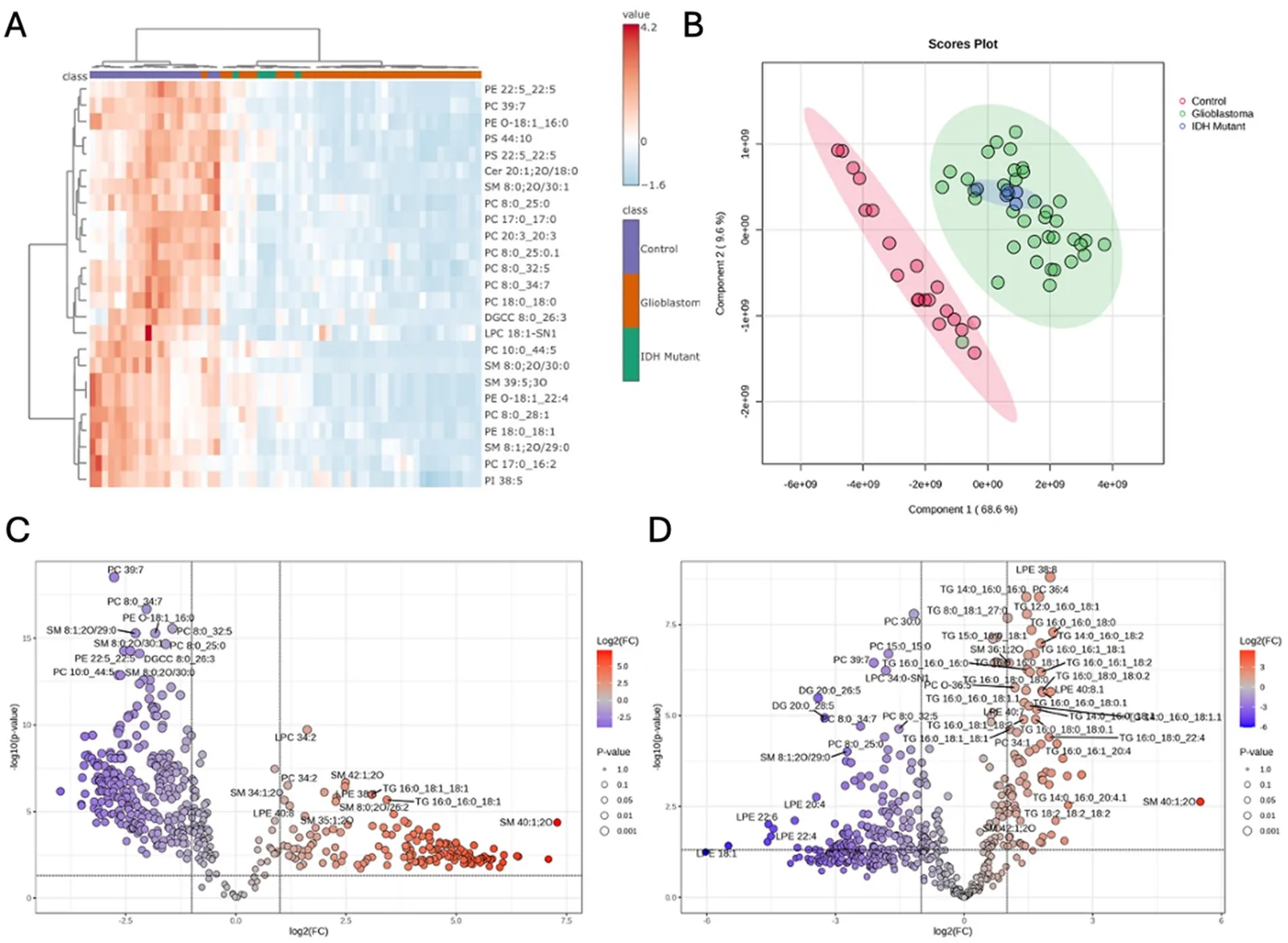
Global lipidomic landscape of high-grade gliomas. (A) Hierarchical clustering heatmap of the top lipid species representing the most variance across control, GBM, and IDH mutant samples. (B) PLS-DA scores plot demonstrating that tumor entities co-cluster together, distinctly separate from healthy brain tissue. (C, D) Volcano plots illustrating significantly altered lipids in GBM (C) and IDH mutant (D) tumors compared to non-neoplastic controls. Significant features include increases of triglyceride species and decreases in phosphatidylcholines, among others.

Lipidomic profiling was next characterized between the three groups. Hierarchical clustering was performed to evaluate the top 25 lipids that best represented the variance of the broader dataset. This demonstrated that while GBM and IDH mutant overlap, they have distinct clustering from the control. (Figure 2A). Partial least squares discriminant analysis was also performed to examine the variance of the lipids across the entire dataset from all three groups. The resulting score plot similarly demonstrates that while GBM and IDH overlap, they have distinct clustering from the control (Figure 2B). This separation was found to be statistically significant and robust, as confirmed by permutation testing (*P* < .001) and five-fold cross-validation (Figure S2). The significantly altered lipids between GBM and control are depicted in a volcano plot, with sphingomyelin 40:1 being amongst those increased in GBM and phosphatidylcholine 39:7 being amongst those decreased (Figure 2C). The significantly altered lipids between IDH and Control are also depicted in a volcano plot, with sphingomyelin 40:1 again being amongst those increased in IDH and lysophosphatidylethanolamine 22:6 being decreased (Figure 2D). The full list of significantly altered metabolites for each comparison is included in Table S3 for GBM and Table S4 for IDH-mutant.

### Machine Learning Offers Robust Diagnostic Utility

**Figure 3.**
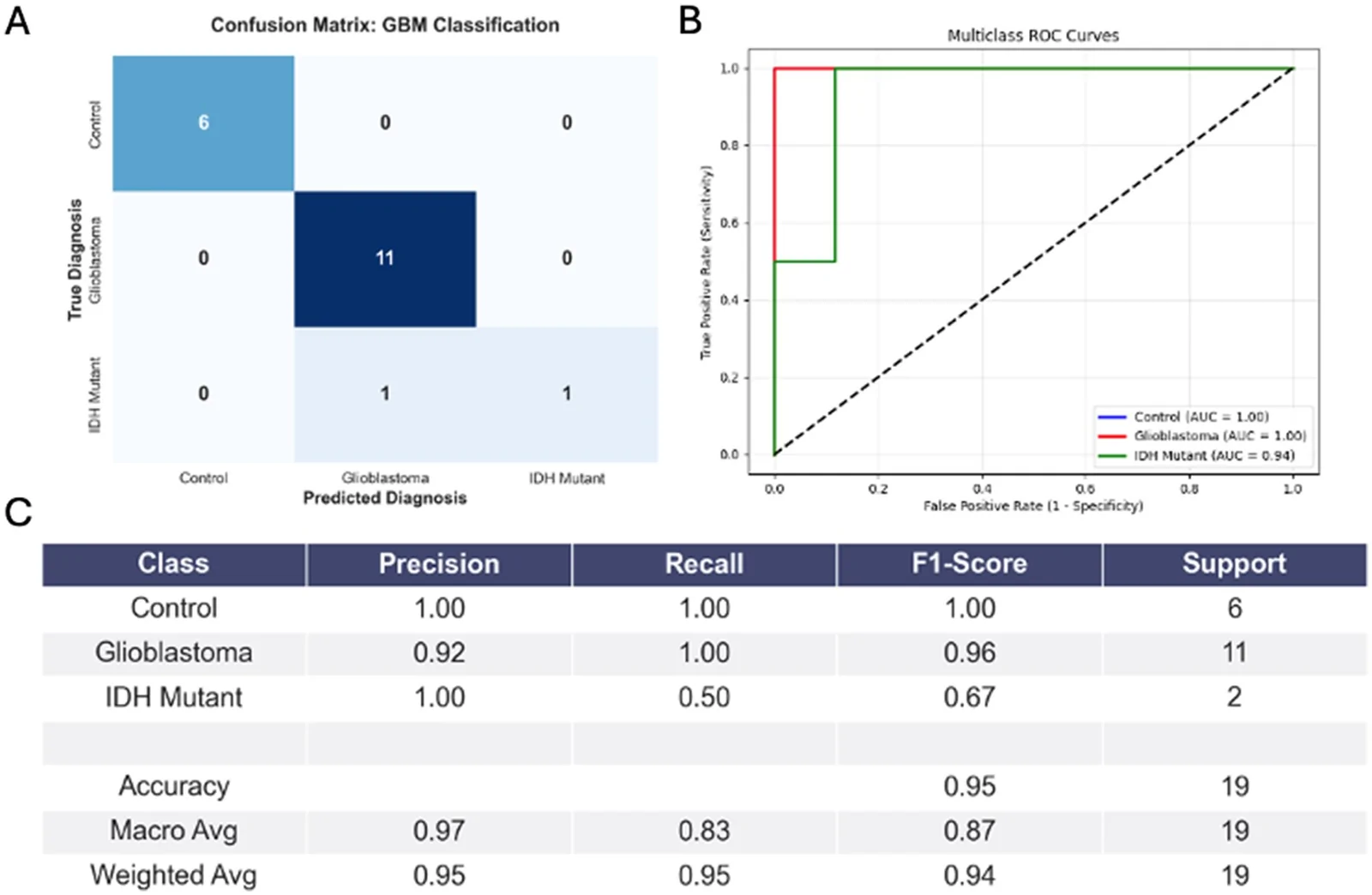
Machine learning classification and diagnostic efficacy. (A) Confusion matrix for a multiclass classifier trained on the top 20 metabolite features, demonstrating high accuracy in distinguishing between cohorts. (B) Multi-class Receiver Operating Characteristic (ROC) curves showing perfect Area Under the Curve (AUC) for control and GBM (AUC = 1.00) and high performance for IDH mutant (AUC = 0.94) classes. (C) Summary table of classification performance metrics, including precision, recall, and F1-scores, achieving an overall test accuracy of 95%.

Individual metabolites can fluctuate, so we sought to determine if a composite signature could provide stronger diagnostic clarity. The top 20 metabolite features identified via PLS-DA variable importance in projection (VIP) scores were extracted to serve as a multivariate biomarker panel. To validate the diagnostic potential of this panel, we trained a multinomial logistic regression classifier using data from 70% (44/63) of the total samples, including 14 control, 27 GBM, and 3 IDH mutant samples. The model was then tested on the remaining 30% (19/63) of the samples, including 6 control, 11 GBM, and 2 IDH mutant samples. Results of the model are depicted in a Confusion matrix with nearly perfect overlap of the Predicted Diagnoses and the True Diagnoses (Figure 3A). Multiclass receiver operating characteristic curves are also depicted, demonstrating areas under the curve (AUC) for control, GBM, and IDH mutant of 1.0, 1.0, and 0.94, respectively (Figure 3B). Summary statistics of the analysis are depicted in Figure 3C, with an overall diagnostic accuracy of the model of 95%.

### Identification of Three Distinct GBM Metabolic Subtypes

**Figure 4.**
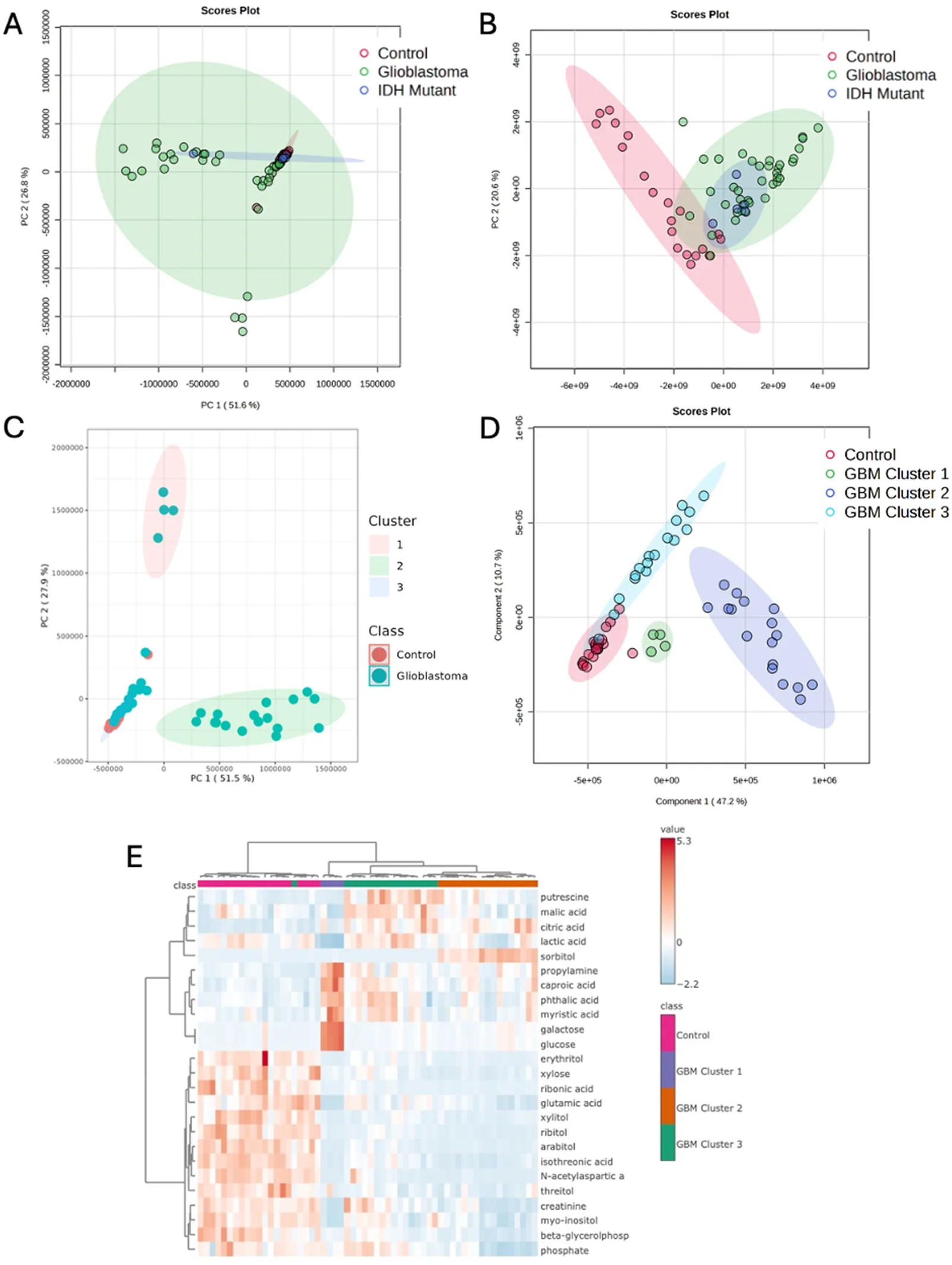
Identification of three metabolic subtypes in GBM. (A, B) Principal Component Analyses (PCA) score plots for the metabolomics dataset (A) and lipidomics dataset (B). (C) K-means analysis identifying three subclusters (Clusters 1–3) within the IDH-wildtype GBM cohort. (D) PLS-DA of the newly-defined three GBM clusters demonstrating visual separation. (E) Hierarchical clustering was performed to evaluate the top 25 metabolites that best represented the variance of these Clusters compared to control, which demonstrated distinct metabolomic signatures.

While GBM metabolomics data is collectively distinct from control tissue, visual inspection of the PLS-DA score plot in Figure 1B suggested that the GBM group may be heterogeneous compared to control and IDH mutant samples. To explore this further, principal component analysis (PCA) was performed on the entire dataset of the three groups (Figure 4A). As this method is unsupervised, it allows for a deeper examination of the heterogeneity of the GBM group, which in this case suggests three possible clusters within GBM (Figure 4A). PCA of the lipidomics data did not suggest any visual clusters (Figure 4B). K-means clustering of the GBM metabolomics data delineated three clusters (Figure 4C), while this same analysis on the lipidomics data did not demonstrate any distinct clusters (data not shown). The three GBM metabolomics clusters (designated Cluster 1, Cluster 2, and Cluster 3) were validated by PCA (Figure 4D), and pairwise permutational multivariate analysis of variance (PERMANOVA) demonstrated that these three clusters were significantly distinct from control samples and significantly distinct from one another (Figure S3C). Hierarchical clustering was performed to evaluate the top 25 lipids that best represented the variance of these Clusters compared to the control, which demonstrated distinct metabolomic signatures (Figure 4E).

### Characterization of the Metabolic Programs of GBM and IDH Tumors

**Figure 5.**
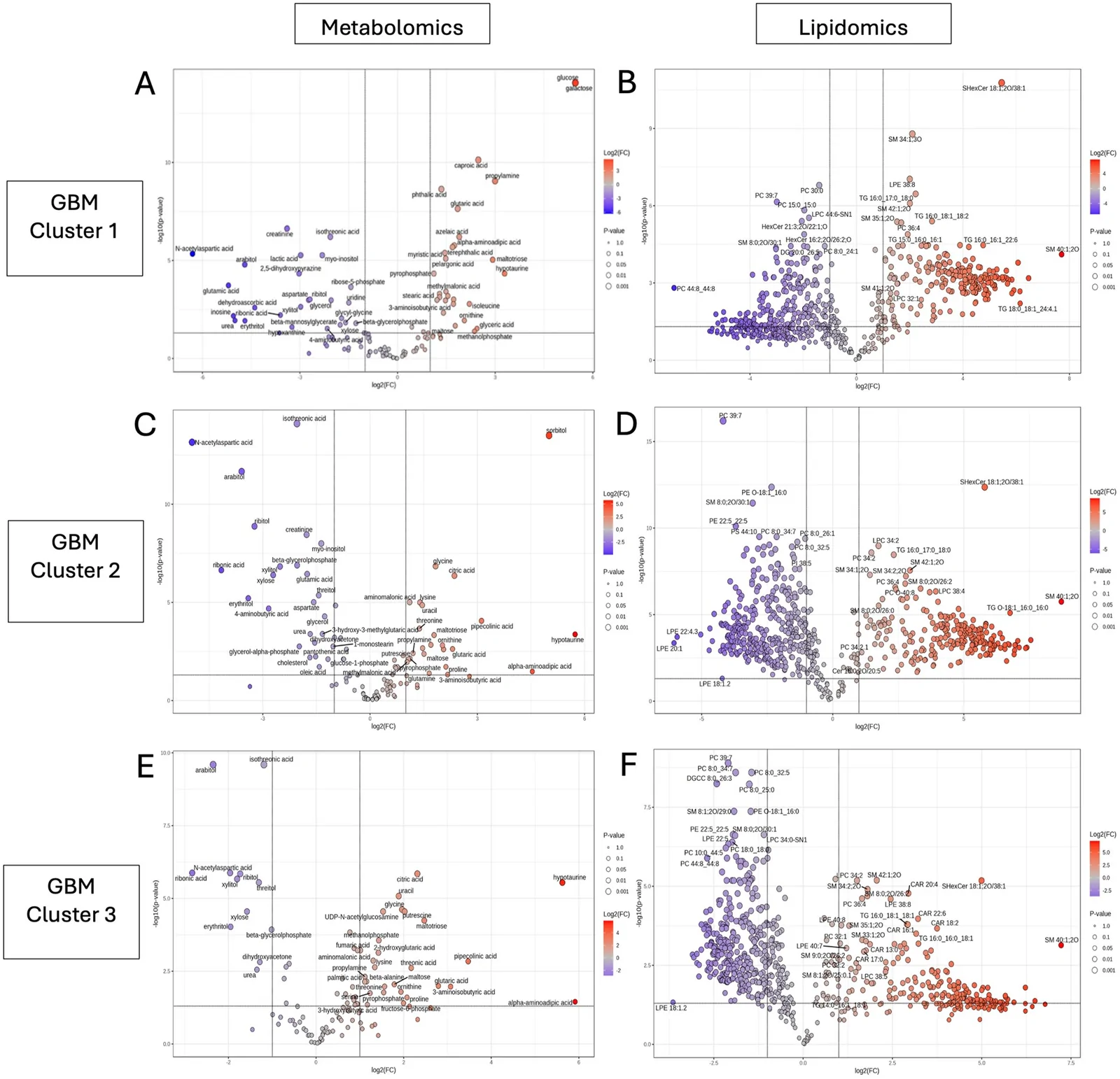
Differential metabolomic and lipidomic landscapes of GBM subtypes and IDH-mutant gliomas. Volcano plots illustrate the differential abundance of metabolites (left panels) and lipid species (right panels) across defined tumor subgroups. Panels display profiles for (A, B) GBM Cluster 1; (C, D) GBM Cluster 2; (E, F) GBM Cluster 3. For all plots, the x-axis represents the log_2_-fold change (log_2_FC), and the y-axis represents the statistical significance as − log_10_(*P*-value). Data points are colored according to regulation direction and magnitude: red/orange indicates upregulation, while blue/purple indicates downregulation. The size of the points corresponds to the significance level. Dashed horizontal and vertical lines denote the thresholds for statistical significance and fold change, respectively. Notable features, such as the accumulation of 2-hydroxyglutaric acid in the IDH-mutant group (G), are annotated.

Now that the three GBM Clusters have been identified, we next examined differences in specific metabolites within each Cluster compared to control samples, as well as within IDH compared to the control samples. Volcano plots demonstrate metabolites and lipids whose abundances are significantly different in each group compared to control (Figure 5A-H), with tables of the detailed results in Supplementary Tables S5-S10. Although each GBM Cluster has distinct metabolomic changes compared to the control, all three demonstrated increased abundance of hypotaurine compared to the control—this increase was also seen in the IDH samples compared to the control (Figure 5A, C, E, and G). IDH samples were found to have a dramatically increased abundance of 2-hydroxyglutaric acid, which is the hallmark metabolite of this tumor type (Figure 5G). Examination of the lipidomics profiles also demonstrated distinct lipidomic signatures within each Cluster; however, all groups demonstrated increased abundances of sphingomyelin 40:1 and SHexCer 18:1 (Figure 5B, D, F, and H).

**Figure 6.**
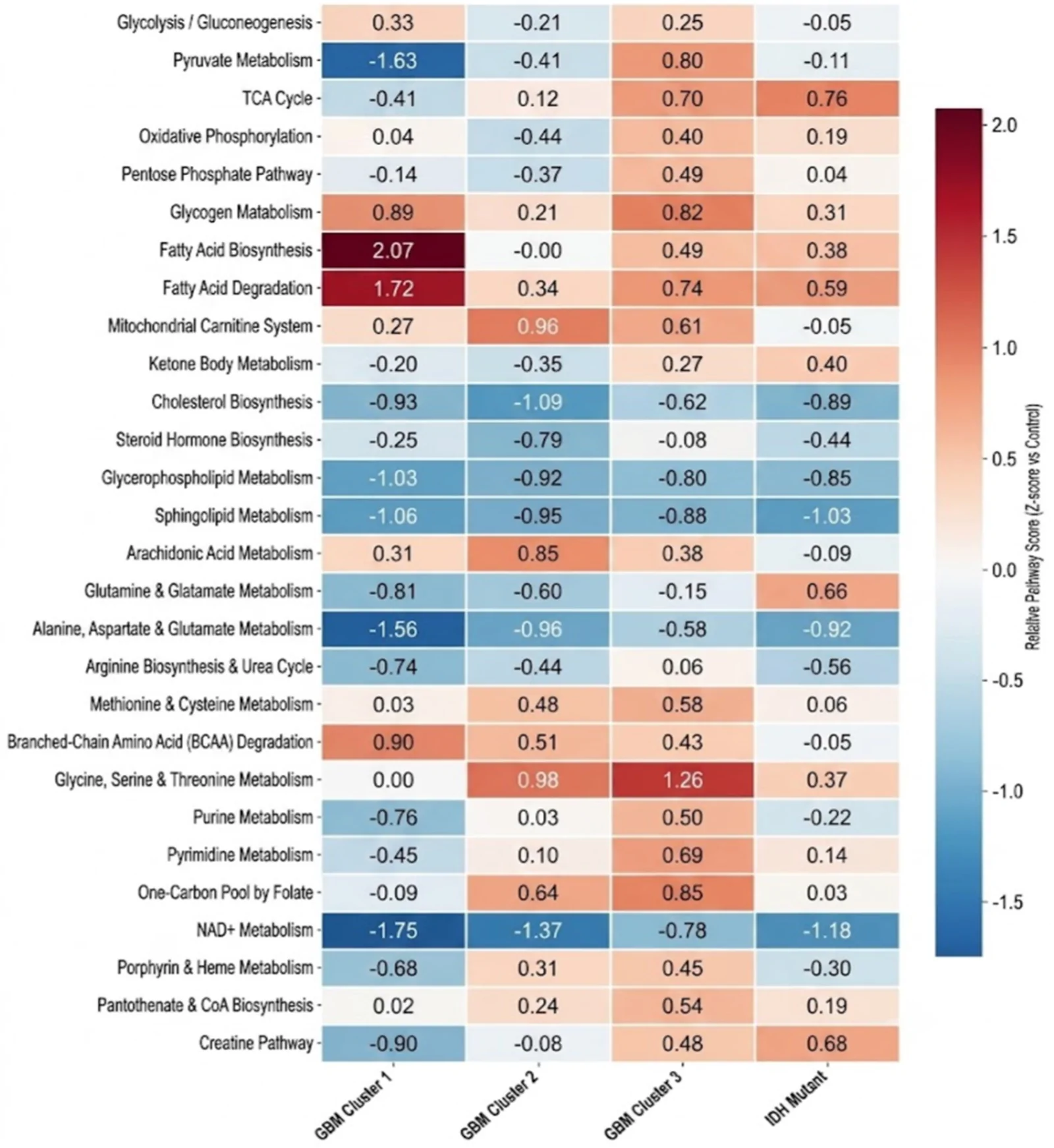
Metabolic pathway alterations across GBM clusters and IDH-mutant gliomas. Heatmap visualization displaying the relative activity of major metabolic pathways (rows) across the identified GBM clusters and IDH-mutant tumors (columns). The color scale represents the Relative Pathway Score, calculated as a Z-score compared to control samples. Red shades indicate upregulated pathways (positive Z-scores), while blue shades indicate downregulated pathways (negative Z-scores). The numeric value within each cell denotes the specific Z-score for the corresponding pathway and tumor subgroup, highlighting distinct metabolic dependencies such as the upregulation of Fatty Acid Biosynthesis in GBM Cluster 1 and glycine, serine & threonine metabolism in GBM Cluster 3.

In order to characterize the functional metabolic architecture of high-grade gliomas, we performed quantitative pathway enrichment analysis mapping of significantly altered metabolites to the Kyoto Encyclopedia of Genes and Genomes (KEGG) reference database. This approach allowed for the calculation of a Relative Pathway Score or Z-score, representing the aggregate regulation of a metabolic module within a tumor group compared to control samples. This analysis demonstrated distinct metabolic programs within each GBM cluster and IDH mutant tumors (Figure 6). GBM Cluster 1 was defined by a uniquely increased abundance of metabolites involved in fatty acid biosynthesis (*Z* = 2.07), fatty acid degradation (*Z* = 1.72), and branched-chain amino acid (BCAA) degradation (*Z* = 0.90), indicating a metabolic profile heavily prioritized toward rapid lipid turnover and dynamic cellular remodeling. GBM Cluster 2 demonstrated uniquely increased abundance of metabolites involved in the mitochondrial carnitine system (*Z* = 0.96) and arachidonic acid metabolism (*Z* = 0.85), reflecting an altered sphingolipid metabolism and complex lipid accumulation profile. GBM Cluster 3 exhibited uniquely increased abundance of pyruvate metabolism (*Z* = 0.80), glycine, serine and threonine metabolism (*Z* = 1.26), purine metabolism (*Z* = 0.50), pyrimidine metabolism (*Z* = 0.69), and one-carbon pool by folate (*Z* = 0.85), highlighting a metabolic program strongly skewed toward anabolism, nucleotide synthesis, and rapid cellular proliferation (Figure 6).

**Figure 7.**
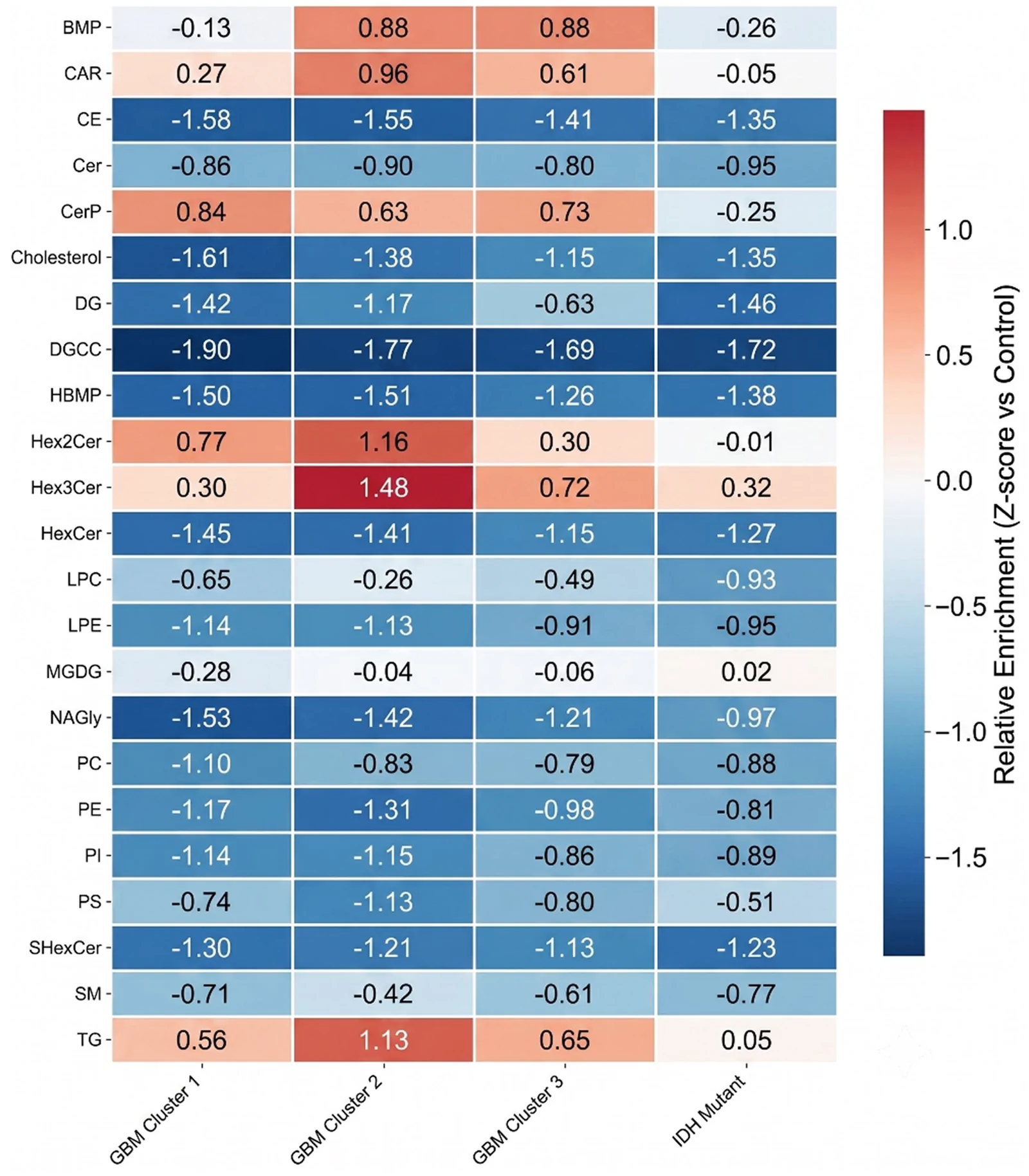
Lipid class enrichment profiles across GBM clusters and IDH-mutant gliomas. Heatmap visualization showing the relative enrichment of distinct lipid classes (rows; see Supplementary Table S11 for full names of the above abbreviations) across GBM subgroups and IDH-mutant tumors (columns). The color scale represents the abundance Z-score relative to control samples, with red indicating enrichment (positive values) and blue indicating depletion (negative values). Numeric values within each cell denote the specific Z-score for the corresponding lipid class and tumor subgroup. The analysis reveals specific lipidomic signatures, such as the marked accumulation of trihexosylceramides (Hex3Cer) and triglycerides (TG) in GBM Cluster 2, contrasted with a widespread depletion of cholesterol and diacylglycerylcarboxy-N-hydroxymethylcholine (DGCC) across all tumor cohorts.

Now that the metabolomic signatures have been characterized, the lipidomics data were subjected to lipid class enrichment analysis (LCEA), wherein each GBM Cluster and IDH were compared to control samples. Lipid classes were defined according to the LIPID MAPS ontology.^14^ Comparison of the overall lipid class profiles between tumor types suggests significant similarity in the lipid modifications in these tumors compared to control samples (Figure 7). This result agrees with prior analyses in Figure 4B demonstrating that the lipidomic signatures of GBM samples do not form distinct clusters. This LCEA demonstrated an increase in triglycerides (TG), ceramides (Cer), and acylcarnitines (CAR) across all GBM clusters; however, the magnitude of these changes is greatest in Cluster 2 (Figure 7).

### Quantitative Analysis of Metabolic and Lipidomic Indices

**Figure 8.**
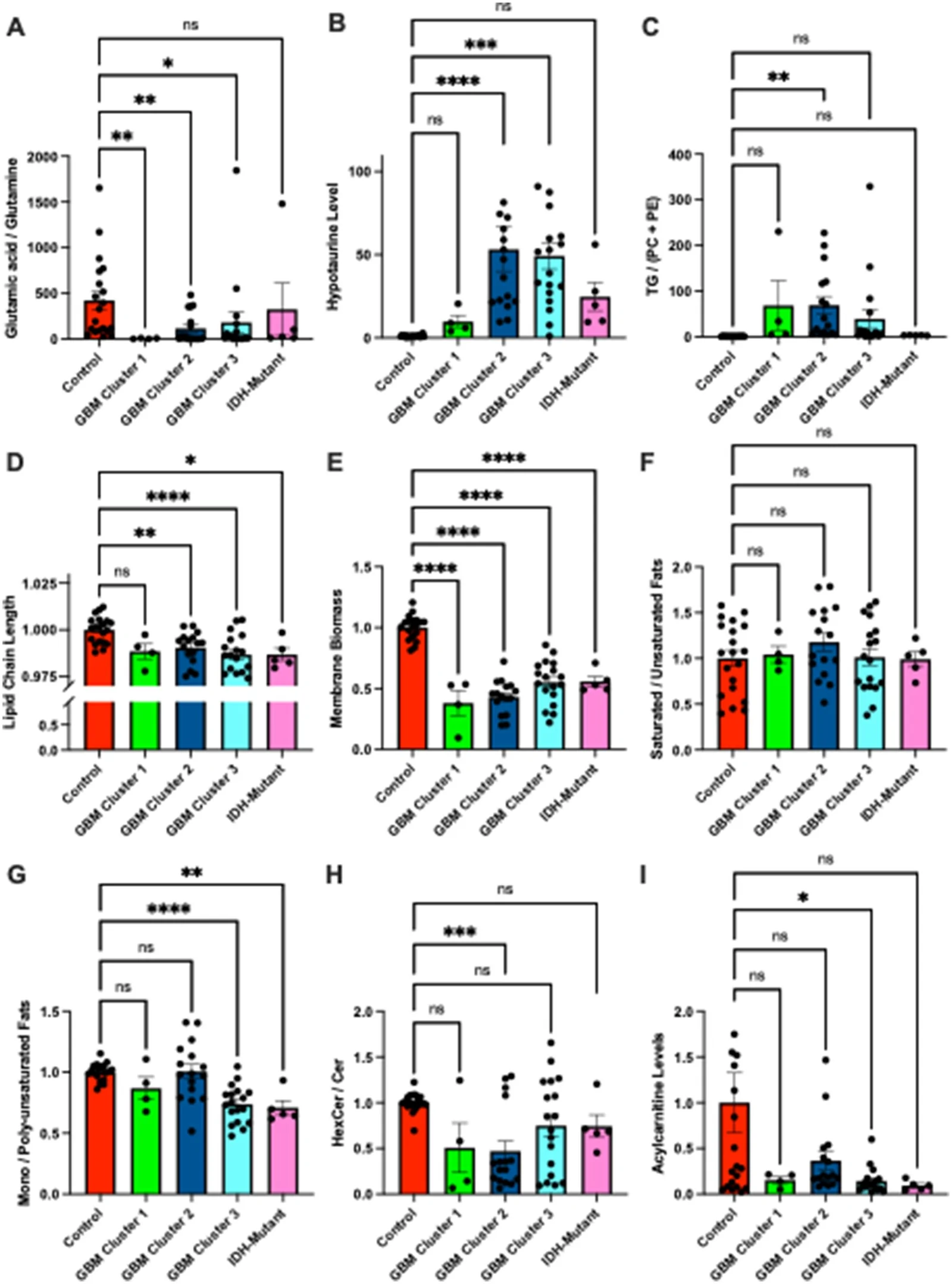
Quantitative analysis of metabolic and lipidomic alterations across GBM subtypes and IDH-mutant tumors. Bar graphs illustrating differential metabolic indices and lipid structural features across Control tissues, distinct GBM Clusters (1–3), and IDH-mutant tumors. (A) Ratio of glutamic acid to glutamine, serving as a proxy for glutaminolysis activity. (B) Relative abundance of hypotaurine, indicative of antioxidant capacity. (C) Energy storage index, calculated as the ratio of triglycerides (TG) to the sum of major membrane phospholipids, phosphatidylcholine (PC) and phosphatidylethanolamine (PE). (D) Average lipid chain length relative to control. (E) Total membrane biomass, representing the aggregate abundance of structural lipids. (F) Membrane saturation index, defined as the ratio of total Saturated fatty acids to Unsaturated fatty acids. (G) Ferroptosis resistance index, measured as the ratio of Monounsaturated fatty acids (MUFA) to polyunsaturated fatty acids (PUFA). (H) Drug resistance marker, calculated as the ratio of hexosylceramides (HexCer) to ceramides (Cer). (I) Mitochondrial stress marker, assessed by the total abundance of acylcarnitines. Data are presented as mean ± SEM with individual biological replicates shown as points. All groups values have been normalized to the respective Control group, which is given a mean value of 1. Statistical significance relative to the Control group (or between bracketed groups) is indicated by asterisks: *P < .05, **P < .01, ***P < .001, ****P < .0001; *ns* = not significant.

Functional analyses were next examined to infer potential novel insights about tumor biology by examining metabolic flux and the structural characteristics of tumor tissues. Quantitative analysis of glutaminolysis revealed that while Control samples maintain a high glutamic acid-to-glutamine ratio, all three GBM clusters demonstrate significantly decreased ratios (Figure 8A). Hypotaurine, a proxy for antioxidant capacity, was significantly increased in all tumor groups but only reached statistical significance for GBM Cluster 2 and Cluster 3 (Figure 8B). Examination of energy storage in the form of triglycerides (TG) revealed a significant increase in TG levels in GBM Cluster 2 when compared to the structural lipids of phosphatidylcholine (PC) and phosphatidylethanolamine (PE) (Figure 8C). All tumor groups had decreased membrane biomass and lipid chain length—though GBM Cluster 1 did not reach significance for lipid chain length (Figure 8D and E). There was no significant difference in the ratio of saturated to unsaturated fatty acids in all groups—a marker of membrane fluidity (Figure 8F). Resistance to ferroptosis has been approximated by examining the ratio of monounsaturated to polyunsaturated fatty acids,^11^ and here we find that GBM Cluster 3 and IDH tumors have a decreased ratio compared to control samples, suggesting that they are susceptible to oxidative lipid damage (Figure 8G). The ratio of hexosylceramide to ceramide approximates the activity of glucosylceramide synthase (GCS) and is a canonical mechanism of drug resistance;^12^ here we demonstrate that this ratio is significantly reduced in GBM Cluster 2 but not the other tumors (Figure 8H). Lastly, acylcarnitine levels are often used as a proxy for mitochondrial stress, as they can accumulate due to defective beta-oxidation or electron transport chain activity.^15^ Here, all tumor groups trended toward a decreased relative abundance of acylcarnitines, but significance was only reached in GBM Cluster 3 (Figure 8I).

## Discussion

Significant efforts are being made to understand the transcriptional programs of high-grade gliomas through single-cell RNA sequencing.^4^ A limitation of this approach is that gene expression hints at a cancer’s *potential*; however, metabolism suggests a cancer’s *action*. A detailed description of the metabolic landscapes of high-grade gliomas is currently lacking. Previous work has primarily focused on isolated metabolic pathways such as glycolysis or glutamine metabolism, or utilized targeted approaches, often missing the comprehensive metabolic and structural lipid landscape that dictates glioma pathophysiology and therapeutic resistance.^16^

Here, we demonstrate that GBM and IDH mutant gliomas have distinct metabolomic and lipidomic profiles compared to control brain tissue. Upon examination of the metabolomic results, we identified a panel of metabolites that are increased solely in GBM compared to controls, including sorbitol and uracil. Metabolites that are specifically increased in IDH-mutant tumors include 2-hydroxyglutarate and alpha-aminoadipic acid, propylamine, and beta-alanine. On the other hand, there are numerous metabolites that are similarly increased in both GBM and IDH-mutant tumors compared to controls, including hypotaurine, glutaric acid, citric acid, pipecolinic acid, maltotriose, and glycine, among others. In particular, the accumulation of citric acid and maltotriose in both tumor types may suggest a convergent reliance on altered TCA cycle flux and alternative carbon sourcing to maintain energy production in the nutrient-poor brain microenvironment.^5^ 2-hydroxyglutarate is well-established as a metabolic driver of the pathogenesis of IDH-mutant tumors and has been previously demonstrated to be increased in IDH-mutant tumors,^17^ the re-demonstration of this result in our work supports the reliability of our findings.

One particularly interesting result from our metabolomics results is the metabolite hypotaurine, which is significantly increased compared to Controls across all three functionally distinct clusters of IDH-wildtype GBM, as well as in IDH-mutant astrocytomas. The ubiquitous accumulation of hypotaurine, rather than being restricted to a single metabolic subtype, suggests that it may act as a critical, convergent oncometabolite driving the progression of diverse high-grade gliomas. The accumulation of this metabolite is potentially as glioma cells upregulate the synthesis of antioxidants.^18^ Once accumulated, hypotaurine actively promotes tumor aggressiveness through multiple mechanisms. It competitively inhibits prolyl hydroxylase domain-2 (PHD2), which prevents the degradation of hypoxia-inducible factors (HIF-1α and HIF-2α) and triggers an aberrant pseudo-hypoxic signaling cascade that enhances tumor proliferation and invasion.^19^ Furthermore, hypotaurine and its biosynthetic enzyme, cysteamine dioxygenase (ADO), are essential for maintaining glioma stem cell phenotypes.^18^ The ADO/hypotaurine axis activates the NF-κB signaling pathway, which subsequently drives the secretion of the chemokine CCL20. This establishes a positive feedforward loop, as elevated CCL20 further stimulates NF-κB to maintain glioma stem cell self-renewal and clonogenicity.^18^ A schematic depicting this mechanism is included in Figure S4. Because this robust metabolic dependency is shared across IDH-mutant tumors and all IDH-wildtype GBM subtypes, targeting the hypotaurine pathway represents a highly promising, subtype-independent therapeutic vulnerability.

Both GBM and IDH-mutant tumors share similar metabolites that have decreased levels, including N-acetylaspartic acid, ribitol, xylitol, aspartate, and urea. N-acetylaspartic acid is a well-established marker of neuronal health,^20^ and its decrease in brain tumor samples likely suggests neuronal loss and displacement by infiltrating tumor cells.^21^ Complementing these metabolomic findings, lipidomic profiling revealed equally striking lipid changes in the tumors. Both GBM and IDH mutant tumors exhibited increased levels of numerous triglycerides and sphingomyelins, while exhibiting similar decreases in phosphatidylcholines, among others. Sphingomyelin 40:1 is a particular lipid species that is highly increased in both tumors; this particular species has not been examined in gliomas, but sphingomyelins have been implicated in the organization of lipid rafts on the plasma membrane and thus regulate cell signaling.^22^

This work identified numerous metabolites that are altered in high-grade gliomas. While metabolites like 2-hydroxyglutarate are already established markers for IDH-mutant gliomas, relying on a single metabolite for IDH-wildtype tumors is challenging due to inherent metabolic heterogeneity. To overcome this challenge, we leveraged a machine learning approach to construct a multivariate biomarker panel. The Multinomial Logistic Regression (MLR) classifier, trained on the top 20 most differential metabolites, achieved a 95% accuracy in distinguishing between GBM, IDH-mutant tumors, and control samples. This algorithm demonstrates that a composite metabolic signature can provide robust diagnostic clarity. As such, in clinical cases wherein there is diagnostic uncertainty in the pathologic evaluation of a tumor biopsy, querying this distinct 20-metabolite panel may serve as a useful adjunctive analysis to arrive at a diagnosis. However, future studies will be needed in larger, multi-center cohorts in order to validate these findings.

Numerous studies have identified subclusters of GBM by examining the transcriptional programs of the tumors. Early studies based on bulk sequencing identified three subtypes, designated as proneural, mesenchymal, and classical.^3^ Subsequent studies utilizing single-cell RNA sequencing have since identified more clusters, and studies are ongoing to examine the functional implications of these different transcriptional profiles.^4^ However, an examination of the metabolic programs of these tumors based on direct metabolomic analyses has been lacking. This study demonstrates a novel finding through the identification of three distinct subclusters of GBM based on metabolomic signatures. Unsupervised clustering of the IDH-wildtype GBM cohort revealed three distinct functional metabolic phenotypes.

Pathway analysis of Cluster 1 demonstrated a striking, concurrent upregulation of both fatty acid biosynthesis (*Z* = 2.07) and fatty acid degradation (*Z* = 1.72) relative to control tissues. While the simultaneous synthesis and oxidation of lipids appear energetically paradoxical, this rapid lipid turnover reflects extreme metabolic flexibility. Tumor cells in the hypoxic, nutrient-deprived core often rely on the continuous remodeling of their lipid pools to maintain membrane integrity and buffer against nutrient fluctuations.^5^ This active cycling suggests that Cluster 1 tumors prioritize dynamic lipid remodeling over stable lipid storage or active one-carbon metabolism. Whether this ‘lipid turnover’ phenotype correlates with specific transcriptional subtypes warrants further investigation.

Cluster 2 exhibited a distinct profile characterized by the heavy accumulation of energy storage and complex structural lipids, specifically driven by high enrichments in triglycerides (*Z* = 1.13) and trihexosylceramides (*Z* = 1.48). This accumulation is structurally confirmed by a significantly elevated energy storage index (ratio of triglycerides to structural membrane phospholipids) (Figure 8C). Notably, despite the accumulation of complex hexosylceramides, Cluster 2 demonstrated a significantly reduced hexosylceramide-to-ceramide (HexCer/Cer) ratio (Figure 8H). The conversion of pro-apoptotic ceramides to stable hexosylceramides via glucosylceramide synthase (GCS) is a well-established mechanism by which cancer cells evade therapy-induced apoptosis.^12^ The significant depletion of this ratio uniquely positions Cluster 2 as having an altered ceramide metabolic flux, which may imply a distinct vulnerability to traditional alkylating agents or targeted ceramidase inhibitors that rely on ceramide accumulation to induce cell death.

Cluster 3 demonstrated a metabolic signature heavily skewed toward anabolic and nucleic acid-supporting pathways, marked by the highest relative scores for glycine, serine, and threonine metabolism (*Z* = 1.26) and one-carbon pool by folate metabolism (*Z* = 0.85). Concurrently, this cluster displayed significant structural lipid alterations that directly impact cell survival. Specifically, Cluster 3 exhibited a significantly decreased ferroptosis resistance index, defined by a reduced ratio of monounsaturated to polyunsaturated fatty acids (MUFA/PUFA) (Figure 8G). Because polyunsaturated fatty acids are the primary requisite substrates for lethal lipid peroxidation, tumors enriched in PUFAs relative to MUFAs are inherently primed for ferroptotic cell death.^11^ Furthermore, Cluster 3 was the only subtype to show a statistically significant depletion in total acylcarnitines (Figure 8I), potentially indicating an impairment or downregulation of mitochondrial fatty acid import in favor of alternative metabolic programs.^15^ Clinically, these structural features suggest that Cluster 3 tumors are hypothesized to be intrinsically primed for therapies designed to induce oxidative stress and trigger ferroptosis. Future in vitro studies utilizing ferroptosis-inducing agents are required to confirm this functional vulnerability.

The identification of these three subclusters of GBM raises interesting questions for future studies; however, limitations of the current work will need to be addressed. The identification of the three subclusters may potentially be due to sampling differences of regions of the tumors since it is known that gliomas have heterogeneous tumor environments—though that may raise a different set of interesting questions. Furthermore, the sample sizes are small, and these results will need to be replicated in larger and potentially multi-institutional studies.

Several limitations of this study must be acknowledged. First, as an exploratory, untargeted profiling study, our findings lack an independent validation cohort. Furthermore, while we identified robust structural and metabolic alterations, we did not perform functional *in vitro* or *in vivo* testing (eg, pharmacologic inhibition of the hypotaurine pathway or ferroptosis induction assays); therefore, the proposed therapeutic vulnerabilities remain hypothesized and require experimental validation. Second, clinical metadata, including survival outcomes and comprehensive genomic/transcriptomic profiles, were unavailable for this cohort, preventing the correlation of our metabolic clusters with patient prognosis or established molecular subtypes. Third, the statistical power of our subgroup analyses is limited by sample size, particularly the IDH-mutant cohort (*n* = 5) and our ability to assess sex as a biological variable. Finally, our non-neoplastic control tissues were derived from patients with traumatic brain injury and epilepsy, conditions known to independently influence brain metabolism and neuroinflammation. Future multi-institutional studies are necessary to address these confounding variables and validate these metabolic subtypes.

## Conclusion

In summary, untargeted metabolomic and lipidomic profiling reveals that IDH-wildtype GBM is not a functional monolith but rather may comprise metabolic subtypes. These functional clusters are defined by unique structural lipid landscapes and metabolic vulnerabilities, such as the intrinsic susceptibility to ferroptosis. Recognizing these active metabolic phenotypes bridges a critical translational gap left by transcriptomics alone. Ultimately, this framework provides a rationale for precision metabolic oncology, wherein metabolic interventions may be tailored to the patient’s GBM subtype. Furthermore, the identification of a 20-metabolite biomarker panel coupled with a machine learning classifier demonstrates the feasibility of using metabolic signatures as a complementary diagnostic tool in cases of diagnostic ambiguity.

## Supplementary Material

vdag199_Supplementary_Data

## Data Availability

All data can be made available upon request.
