# Supplementary material for: Metabolomic and lipidomic profiling reveals distinct subtypes of glioblastoma IDH-wildtype and shared metabolic features with astrocytoma IDH-mutant grade 4
Source: Neurooncol Adv. 2026 Sep 2;8(1):vdag199. doi: 10.1093/noajnl/vdag199 (PMC13548785; doi:10.1093/noajnl/vdag199)
Supplement: Supplementary file 1 [file vdag199_supplementary_data.zip › Revised_Supplementary Data.docx]

Supplementary Data

**Supplementary Figure S1: Validation of the Global Metabolomics PLS-DA Model.** (A) 5-fold cross-validation performance evaluating Accuracy, R2, and Q2 across 5 components. The optimal number of components is indicated by the red asterisk. (B) Permutation testing (n = 1000 permutations) demonstrating model robustness (p < 0.001).

**Supplementary Figure S2: Validation of the Global Lipidomics PLS-DA Model.** (A) 5-fold cross-validation performance evaluating Accuracy, R2, and Q2 across 5 components. The optimal number of components is indicated by the red asterisk. (B) Permutation testing (n = 1000 permutations) demonstrating model robustness (p < 0.001)

**Supplementary Figure S3: Unsupervised clustering demonstrates significant differences in the metabolomic profiles between GBM clusters.** (A) PCA score plot demonstrating that unsupervised analysis of the metabolomics data variation demonstrates significant differences between Clusters as quantified in the table below with p-values denoted as “pval”. (B) PCA score plot demonstrating that unsupervised analysis of the lipidomics data variation largely demonstrates overlap of the three Clusters, with less robust separation compared to what was observed for the metabolomics data.

**Supplementary Figure S4: Mechanisms of hypotaurine-mediated glioma progression.** The schematic illustrates how the upregulation of antioxidant synthesis leads to the accumulation of hypotaurine, which subsequently acts as an oncometabolite driving glioma aggressiveness through two distinct signaling branches. On the left, hypotaurine competitively inhibits prolyl hydroxylase domain-2 (PHD2), preventing the degradation of hypoxia-inducible factors (HIF-1α and HIF-2α). This initiates an aberrant pseudo-hypoxic signaling cascade that enhances tumor proliferation and invasion. On the right, the cysteamine dioxygenase (ADO)/hypotaurine axis activates the NF-κB signaling pathway to drive the secretion of the chemokine. The increased secretion of CCL20 establishes a positive feedforward loop that further stimulates NF-κB signaling, which is essential for maintaining glioma stem cell (GSC) self-renewal and clonogenicity (Shen et al., 2021).
